# Supplementary material for: Identification of a Biomarker Combination for Survival Stratification in pStage II/III Gastric Cancer after Curative Resection
Source: Cancers (Basel). 2022 Sep 12;14(18):4427. doi: 10.3390/cancers14184427 (PMC9497152; doi:10.3390/cancers14184427)
Supplement: Supplementary file 1 [file cancers-14-04427-s001.zip › cancers-1870088-Supplementary Figure.pdf]

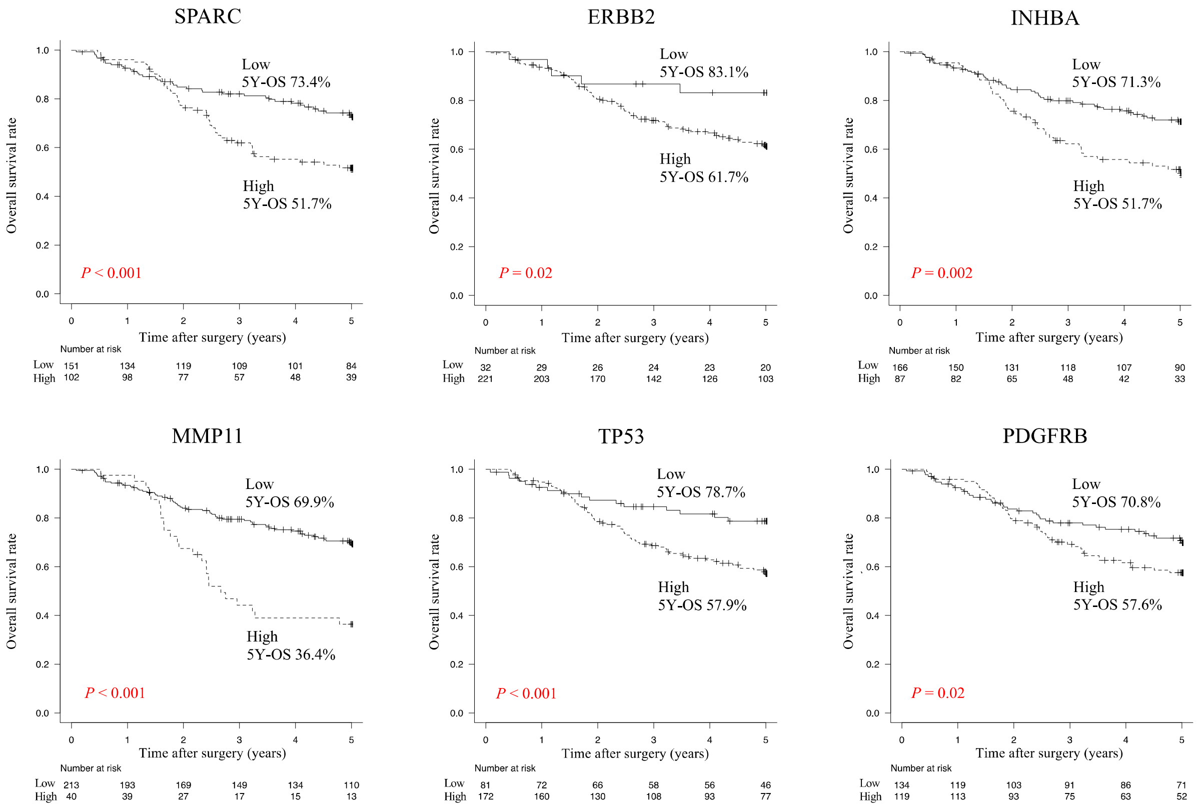

Figure S1: Overall survival rate (OS) of stage II/III gastric cancer (GC) patients based on the expression of each candidate's survival risk stratification marker in 255 patients.

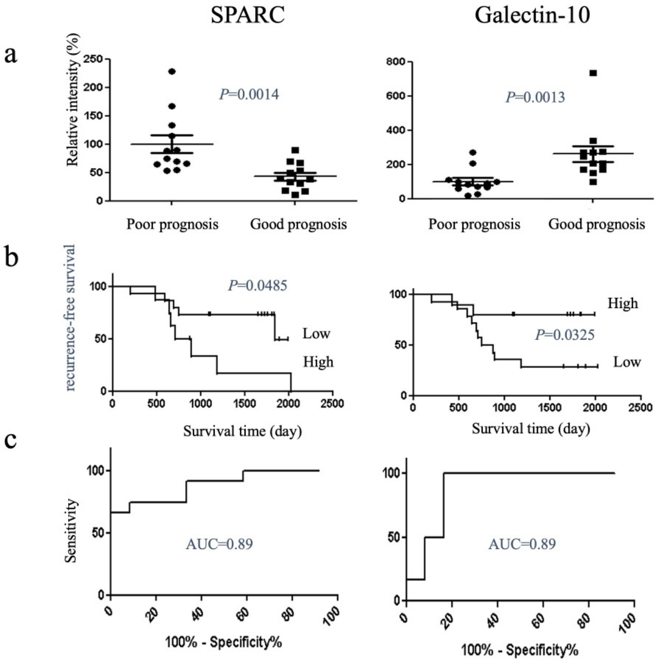

Figure S2: Results of the evaluation of candidate survival risk stratification markers by proteomic analysis using liquid chromatography-mass spectrometry (LC-MS)/MS.

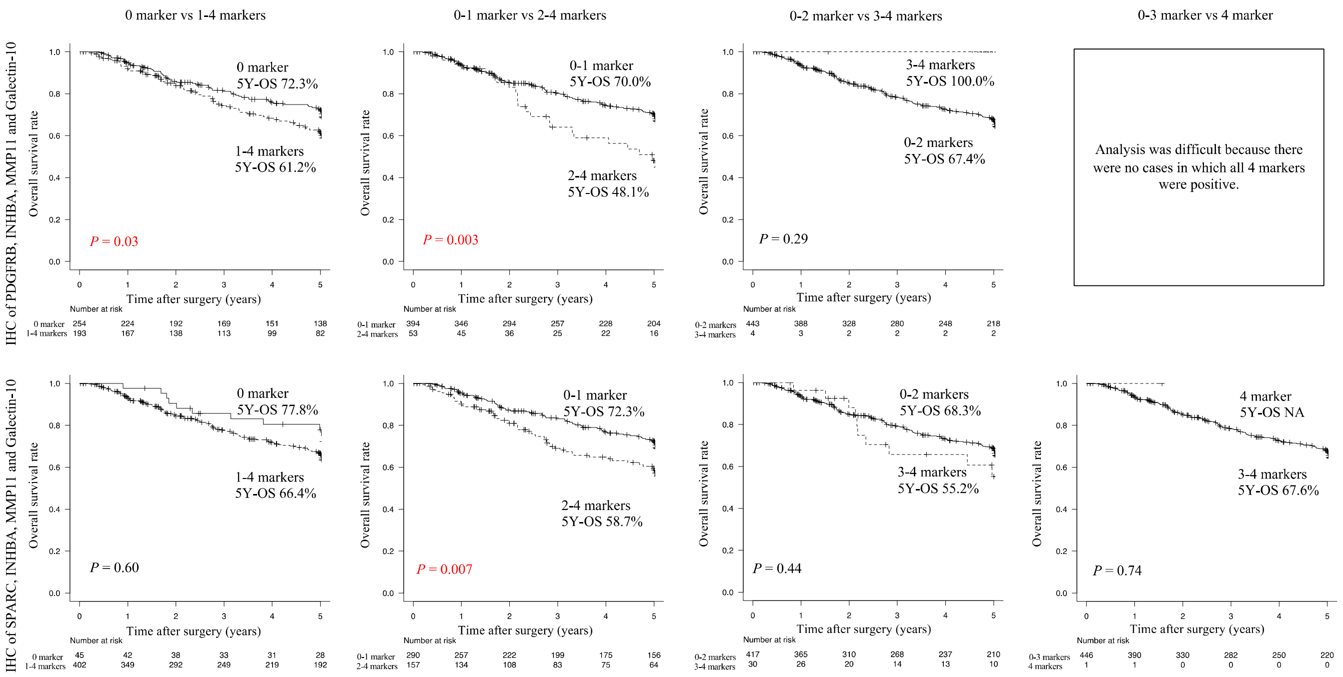

Figure S3: Overall survival rate (OS) of stage II/III gastric cancer (GC) patients stratified according to the combination of survival risk stratification marker expression based on the number of positive markers.

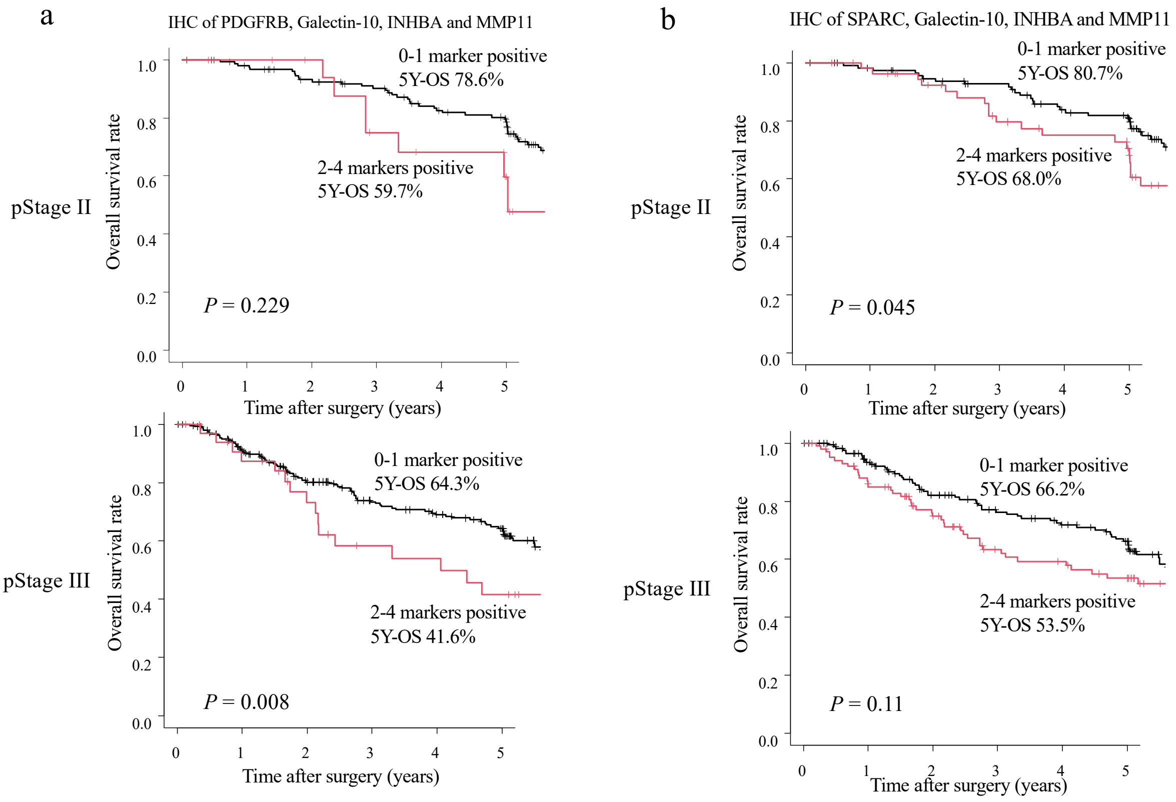

Figure S4: Overall survival (OS) of stage II/III gastric cancer (GC) patients according to the expression of a combination of risk stratification markers.
