# Supplementary material for: Identification of a Biomarker Combination for Survival Stratification in pStage II/III Gastric Cancer after Curative Resection
Source: Cancers (Basel). 2022 Sep 12;14(18):4427. doi: 10.3390/cancers14184427 (PMC9497152; doi:10.3390/cancers14184427)
Supplement: Supplementary file 1 [file cancers-14-04427-s001.zip › cancers-1870088-Supplementary Tables.pdf]

Supplementary Table S1 Univariate and multivariate analyses of clinicopathological factors and Galectin-10 expression for overall survival.

Factors		Number of patients	Univariate HR	Univariate 95%CI	P-Value	Multivariate HR	Multivariate 95%CI	P-Value
Age	≦65	217	1			1		
	＞65	230	1.42	1.08-1.88	**0.014**	1.40	1.06-1.85	**0.019**
Gender	Female	134	1					
	Male	313	1.12	0.82-1.51	0.482			
Histological type	Well/Moderately	193	1					
	Poorly	254	1.07	0.81-1.41	0.643			
Lymphatic invasion	(-)	132	1					
	(+)	315	0.96	0.71-1.29	0.780			
Venous invasion	(-)	128	1					
	(+)	319	1.26	0.93-1.71	0.138			
pStage	II	170	1			1		
	III	277	1.46	1.10-1.94	**0.009**	1.49	1.12-1.98	**0.006**
Galectin-10	Low	364	1			1		
	High	83	1.48	1.06-2.07	**0.021**	1.45	1.04-2.03	**0.030**

p, pathological; Well, Well differentiated adenocarcinoma; Moderately, Moderately differentiated adenocarcinoma; Poorly, Poorly differentiated adenocarcinoma

Supplementary Table S2 Clinicopathological data between Galectin-10 high expression group and low expression groups.

Variables	All patients	Galectin-10		P-value
	(N = 447)	High (N = 83)	Low (N = 364)	
Age	≦65	30 (36.1)	187 (51.4)	**0.015**
	＞65	53 (63.9)	177 (48.6)	
Gender	Male	59 (71.1)	254 (69.8)	0.895
	Female	24 (28.9)	110 (30.2)	
Histological type	Well/Moderately	44 (53.0)	149 (40.9)	0.05
	Poorly	39 (47.0)	215 (59.1)	
Lymphatic invasion	(-)	25 (30.1)	107 (29.4)	0.895
	(+)	58 (69.9)	257 (70.6)	
Venous invasion	(-)	21 (25.3)	107 (29.4)	0.503
	(+)	62 (74.7)	257 (70.6)	
pStage	II	33 (39.8)	137 (37.6)	0.709
	III	50 (60.2)	227 (62.4)	

p, pathological; Well, Well differentiated adenocarcinoma; Moderately, Moderately differentiated adenocarcinoma; Poorly, Poorly differentiated adenocarcinoma

Supplementary Table S3 ROC analysis of the combination of survival risk-stratification marker expression.

IHC of PDGFRB, INHBA, MMP11, and Galectin-10	AUC	95% CI
0 marker vs 1-4 markers	0.519	0.473 - 0.565
0-1 marker vs 2-4 markers	0.515	0.485 - 0.545
0-2 markers vs 3-4 markers	0.504	0.495 - 0.512
0-3 markers vs 4 markers	-	-

IHC of SPARC, INHBA, MMP11, and Galectin-10	AUC	95% CI
0 marker vs 1-4 markers	0.523	0.494 - 0.551
0-1 marker vs 2-4 markers	0.527	0.482 - 0.571
0-2 markers vs 3-4 markers	0.509	0.486 - 0.532
0-3 markers vs 4 markers	0.502	0.498 - 0.506

IHC, Immunohistochemistry; AUC, area under curve; CI, confidence interval

Supplementary Table S4 Clinicopathological data between risk stratification markers high group and low groups.

Variables	All patients (N = 447)	Risk stratification by PDGFRB, MMP11, INHBAG, and Galectin-10		P-value
		High (2-4 markers)	Low (0-1 marker)	
Age	≦65	21 (39.6)	196 (49.7)	0.189
	＞65	32 (60.4)	198 (50.3)	
Gender	Male	42 (79.2)	271 (68.8)	0.150
	Female	11 (20.8)	123 (31.2)	
Histological type	Well/Moderately	34 (64.2)	159 (40.4)	**0.002**
	Poorly	19 (35.8)	235 (59.6)	
Lymphatic invasion	(-)	13 (24.5)	119 (30.2)	0.428
	(+)	40 (75.5)	275 (69.8)	
Venous invasion	(-)	8 (15.1)	120 (30.5)	**0.023**
	(+)	45 (84.9)	274 (69.5)	
pStage	II	19 (35.8)	151 (38.3)	0.765
	III	34 (64.2)	243 (61.7)	

p, pathological; Well, Well differentiated adenocarcinoma; Moderately, Moderately differentiated adenocarcinoma; Poorly, Poorly differentiated adenocarcinoma
